# Screening for Acute HIV Infections and Estimating HIV Incidence among Female Sex Workers from Low-Grade Venues in Guangxi, China

**DOI:** 10.1371/journal.pone.0099522

**Published:** 2014-06-11

**Authors:** Jianjun Li, Hongman Zhang, Zhiyong Shen, Yuejiao Zhou, Ningye Fang, Lu Wang, Bin Wang, Jiangwei Wang, Zhenzhu Tang

**Affiliations:** 1 Guangxi Central Laboratory of HIV/AIDS Confirmation, Institute of HIV/AIDS Prevention and Control, Guangxi Center for Disease Control and Prevention, Nanning, China; 2 Institute of HIV/AIDS Prevention and Control, Guangxi Center for Disease Control and Prevention, Nanning, China; 3 Guangxi Center for Disease Control and Prevention, Nanning, China; The University of Hong Kong, Hong Kong

## Abstract

**Background:**

Guangxi has become one of the provinces with the most severe HIV-1 epidemic in China, where heterosexual contact is the dominant transmission route. However, data of acute HIV infections and HIV incidence among female sex workers (FSWs) from low-grade venues are scant.

**Methods:**

A cross-sectional survey was performed among FSWs from low-grade venues in Guangxi. HIV antibody screening was performed by rapid testing (RT). HIV antibody–negative specimens were screened by pooled nucleic acid amplification testing (NAAT) for acute HIV infections. HIV antibody-positive specimens were further analyzed by Western blot (WB), followed by an HIV-1 BED capture enzyme immunoassay (BED-CEIA) to identify the recent infections. HIV-1 incidence was estimated by the data of pooled NAAT and BED-CEIA, respectively.

**Results:**

A total of 7936 FSWs were recruited and answered the questionnaires. We successfully collected the blood samples from 6469 (81.5%) participants, of which 139 (2.1%) were HIV antibody–positive and 6330 (97.9%) were HIV antibody-negative by RT. With pooled NAAT, 7 cases were found to be HIV RNA positive, representing an additional 5.0% of HIV-infected persons and an estimated HIV incidence of 1.45 (95% CI: 1.17–1.76) per 100 person years. There were 137 positive and 2 indeterminate by WB, of which 124 (90.5%) positive specimens were subjected to BED-CEIA testing identifying 28 recent infections. The HIV incidence determined by BED-CEIA testing was 1.04 (95% CI: 0.65–1.43) per 100 person years. The overall prevalence of HIV among FSWs from low-grade venues in Guangxi was 2.2% (95% CI: 1.9–2.6).

**Conclusions:**

We found that the addition of HIV RNA screening to routine HIV antibody testing significantly improved the detection of HIV infection among FSWs from low-grade venues in Guangxi. Our findings also provided the useful baseline data of HIV incidence among this population for targeting local HIV prevention, intervention, monitoring and treatment.

## Introduction

Heterosexual transmission through commercial sex was recognized as one of the main modes of HIV transmission in China [1]–[3]. Female sex workers (FSWs) were believed to be a “core population”, whose clients were likely to serve as a bridge population to transmit HIV to the general population [4]. It was estimated that there were 1.8–3.8 million FSWs in China [3]. The HIV prevalence of FSWs between 1996 and 2010 in China remained relatively low and stable in a range of 0–10.3% (median = 0.6%), except for several areas of higher prevalence in Yunnan and Guangxi [5]. The size of FSWs population and the HIV prevalence among them were believed to be important factors in determining the spread of HIV epidemic from FSWs to the general population.

Guangxi is located in southern China and has a population of 50 million. It is one of the regions hardest hit by HIV in China and has witnessed an alarming HIV epidemic by heterosexual transmission [6]. Some surveys showed that the HIV prevalence of FSWs in Guangxi was 2.3% in 2005 [7], 0.8% in 2007 [8], and 1.0% in 2010 [9]. The HIV prevalence was significantly higher among FSWs over 40 years old, especially those who worked in small commercial sex venues or on the street, being divorced or widowed, or having no formal schooling [9]. The most prevalent subtype of HIV-1 strain in sexually transmitted infections in Yunnan was first reported to be CRF01_AE in 2006 [10]. Recently, in a study by Li et al. (2013) in collaboration with our center, the CRF01_AE has also been identified as the dominant subtype of current HIV epidemic in heterosexual transmission populations in Guangxi [11]. However, the data of acute HIV infection and HIV incidence among FSWs from low-grade venues are scant.

Currently, three types of HIV antibody screening test routinely used in China are Enzyme-linked immunosorbent assay (ELISA, third generation IgM/IgG-sensitive assay), rapid testing (RT) and chemiluminescence assay [12]. Despite the high sensitivity of these HIV antibody assays, there existed a “window period” during which individuals with the acute HIV infection had negative results by HIV antibody tests [13]. This window period could range from weeks to months depending on the type of tests applied and the differences between the infected individuals, but it could become shorter by testing HIV-1 RNA in plasma or sera. Indeed, an HIV RNA test with sensitivity to detect 50 copies/mL can identify HIV infection approximately 6 to 11 days earlier than does an IgM/IgG-sensitive HIV antibody test and 26 to 31 days earlier than does an IgG-sensitive HIV antibody test [14]. In addition, a few studies showed that an HIV RNA screening strategy incorporating multistage specimen pooling was feasible and cost-effective for detecting acute HIV infection [15]–[18].

HIV-1 incidence can be determined by the traditional cohort method [19] or laboratory assays, such as detection of p24 [20], HIV-1 RNA pooling [16] and BED-CEIA [21], [22]. As cohort studies estimating HIV-1 incidence in developing countries often ended with low follow-up rates and were very expensive [16], alternative or new methods are needed. While BED-CEIA was used in estimation and surveillance of HIV-1 incidence in resource-limited countries [23], HIV-1 RNA pooling appeared to be more sensitive and cost saving than individual testing for p24 antigen [16].

In this study, a cross-sectional survey was performed among FSWs from low-grade venues in Guangxi for HIV infection. This was the first large-scale cross-sectional survey in China utilizing RT for HIV screening, pooled NAAT for detecting acute HIV infections, and BED-CEIA for identifying recent HIV infections. We aimed to elucidate the HIV prevalence and incidence among the FSWs, who are the population at high risk for HIV infections. This study will be helpful for further investigation into the latest trends of HIV infection among FSWs and decision making towards scientific prevention, intervention, and treatment of HIV infections.

## Materials and Methods

### Ethics Statement

The study protocol was approved by the Ethics Review Committee of Guangxi Center for Disease Control and Prevention. Written informed consent was obtained from each participant prior to the interview and sample collection.

### Study Design and Participants

From September to December 2011, a cross-sectional survey was carried out among FSWs from low-grade venues in14 cities and 50 counties in Guangxi. The FSWs from low-grade venues were defined as those who, aged 18 and above, offered sex (vaginal, oral, anal) to men for less than $8 per client in small hotels, rented houses, hair/beauty salons, and restaurants, or streetwalkers. In each target city/county, the low-grade venues were mapped and randomly selected. The participants were recruited to reach at least 200 in each target city and 100 in each target county.

The eligible participants underwent an anonymous face-to-face interview and answered a standardized questionnaire focusing on general demographic information, knowledge of HIV, age, ethnicity, occupation, income, use of condoms, types of intercourse, and substance used. All participants were informed of the new HIV testing protocol whereby HIV antibody-negative specimens were tested for HIV RNA by pooled NAAT and HIV antibody-positive specimens were analyzed for recent infections by BED-CEIA testing. All HIV tests were voluntary. The FSWs then underwent standard pretest risk assessment and risk-reduction counseling provided by the counselors and clinicians who have received training on HIV RNA testing. After the interview, 5 mL blood was collected into sodium EDTA-coated tubes and sent to the laboratory of Center for Disease Control and Prevention (CDC) within 6 h.

### Blood Processing

In the laboratory of CDC in each target city/county, the blood samples were centrifuged at 1000 g for 15 minutes at room temperature. HIV antibodies were screened by RT immediately. The plasma was distributed into 2 small tubes and stored at ≤−20°C temporarily. All plasma tubes were shipped to Guangxi Central Laboratory of HIV/AIDS confirmation and stored at −80°Cfor further analyses.

### HIV Antibody Screening

HIV antibody screening of all specimens was performed by using an HIV-antibody rapid testing assay (Intec, Xiamen, China). When a positive reaction was observed, two additional assays including the original assay plus a different assay (Determine HIV-1/2, Abbott, Japan) were conducted in parallel. If both tests were positive or the results were discordant, the specimens were then shipped to Guangxi Central Laboratory of HIV/AIDS confirmation for further analysis.

### Pooled NAAT and Estimating Incidence

From all participants whose HIV antibody results were negative, an aliquot of plasma was pooled for HIV RNA testing using the multistage pooling scheme modified from a protocol described by Quinn TC et al. [16] and National Guideline for Detection of HIV/AIDS (2009 revision) [12]. Briefly, we used a 2-stage pooling scheme with a master pool comprised of 50 specimens. A 130 µL aliquot from each of 10 specimens were mixed to make an intermediate pool. Then a 210 µL aliquot from each of 5 intermediate pools were mixed to generate a master pool. All individual plasma and pools were stored at −80°C. The HIV-1 real-time PCR testing technology combines the extraction of total nucleic acids on a Cobas AmpliPrep (CAP) with real-time PCR run on a Cobas TaqMan analyzer (CTM) using the Roche COBAS AmpliPrep/COBAS TaqMan HIV-1 Assays, V 2.0 (Roche Molecular Systems, Branchburg, New Jersey, USA). Positive and negative controls were included in each run.

**Figure 1:**
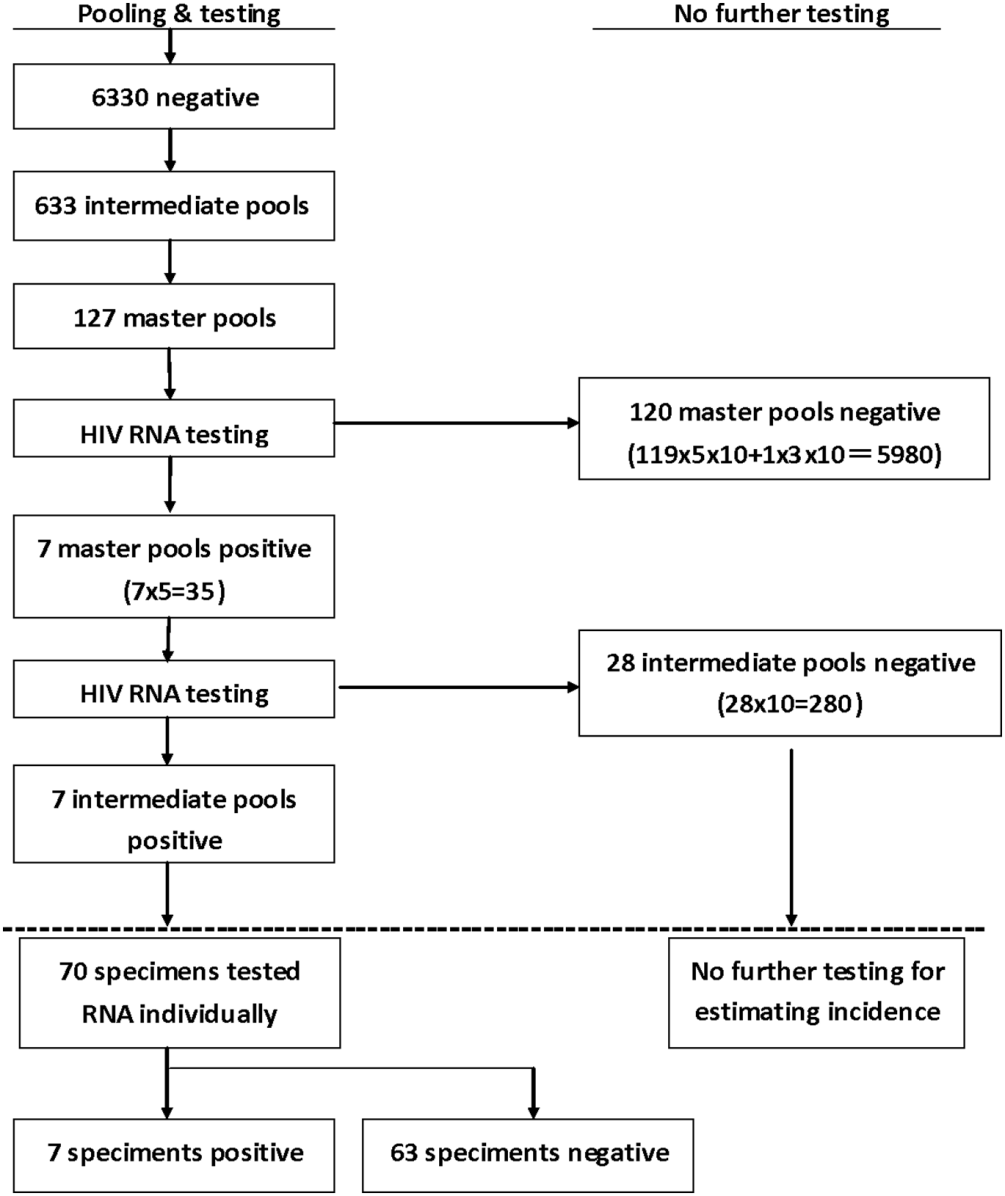
Multistage Pooling Strategy for Screening HIV-1 RNA by the Pooled NAAT. The numbers of pooled samples, the numbers of different pools and the numbers of different RNA results were shown in the figure. HIV incidence was calculated using equation: incidence rate  = 
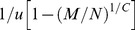
, where *µ* = 28 days, *M* = 5980+280 = 6260, *N* = 6330, *C* = 10.

The HIV-1 RNA pooling algorithm was shown in Figure 1. HIV-1 RNA was first tested in the master pools, then in the intermediated pools and finally in the individual samples. If a master pool or an intermediated pool was negative, no further testing was needed. All specimens in the positive intermediate pools were tested individually.

The maximum likelihood estimator of the incidence rate based on the pooled RNA testing can be obtained from the following formula [24]: 
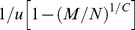
, where *N* is the total number of individual samples that were pooled, *M* is the total number of individual samples in negative pools, *C* is the number of individual samples in each positive pool, and *u* is the duration of RNA positivity prior to seroconversion estimated at 28 days [13], [24]. The 95% confidence intervals (CI) for the incidence rates were also calculated [24]. As *u* is expressed in days (28 days), the calculated incidence from the formula is expressed as a rate per day, so it would be multiplied by 365 for converting to yearly incidence.

### Follow-up and Confirmatory Testing

The participants negative for HIV antibody but positive for HIV RNA were notified for a follow-up and the blood samples would be drawn in 3 months for further test. The 4^th^ enzyme-linked immunosorbent assay (Biomerieux, France) and Western blot (MP Diagnostics, Singapore) were performed on follow-up specimens to document the seroconversion.

### BED-CEIA and Estimating Incidence

Due to potential misclassification by BED-CEIA [25], we firstly excluded specimens from the participants with a history of antiretroviral treatment (ART) or diagnosis of HIV infections for more than 1 year. The remaining samples were tested for recent infections with BED-CEIA. Controls (negative, strongly positive and weakly positive) and calibrators were tested together with the samples using BED-CEIA assay (Calypte Biomedical Corporation-Oregon, USA) according to the manufacturer’s instructions [26]. The crude BED-CEIA estimated HIV incidence was calculated using the US CDC recommended formula [23]: 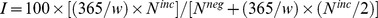, where w is the window period (155 days), *N^inc^* is the number of recent HIV infections as determined by BED-CEIA, and *N^neg^* is the total number of HIV-seronegative subjects. The 95% confidence intervals (CI) for BED-CEIA estimated incidence were calculated by: 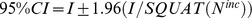 [23]. Exact 95% CI were calculated for HIV incidence based on the Poisson distribution.

### Individual NAAT

Specimens with positive RT but negative or indeterminate WB results were tested with NAAT individually without polling.

### Statistical Analysis

Individuals for whom the RT test was negative but RNA test was positive were defined as having acute HIV infection. Those for whom both the RT test and the RNA test were negative were considered to be free of HIV infection. Descriptive statistics were generated using SPSS version 17.0 (SPSS, Chicago, IL).

## Results

**Figure 2:**
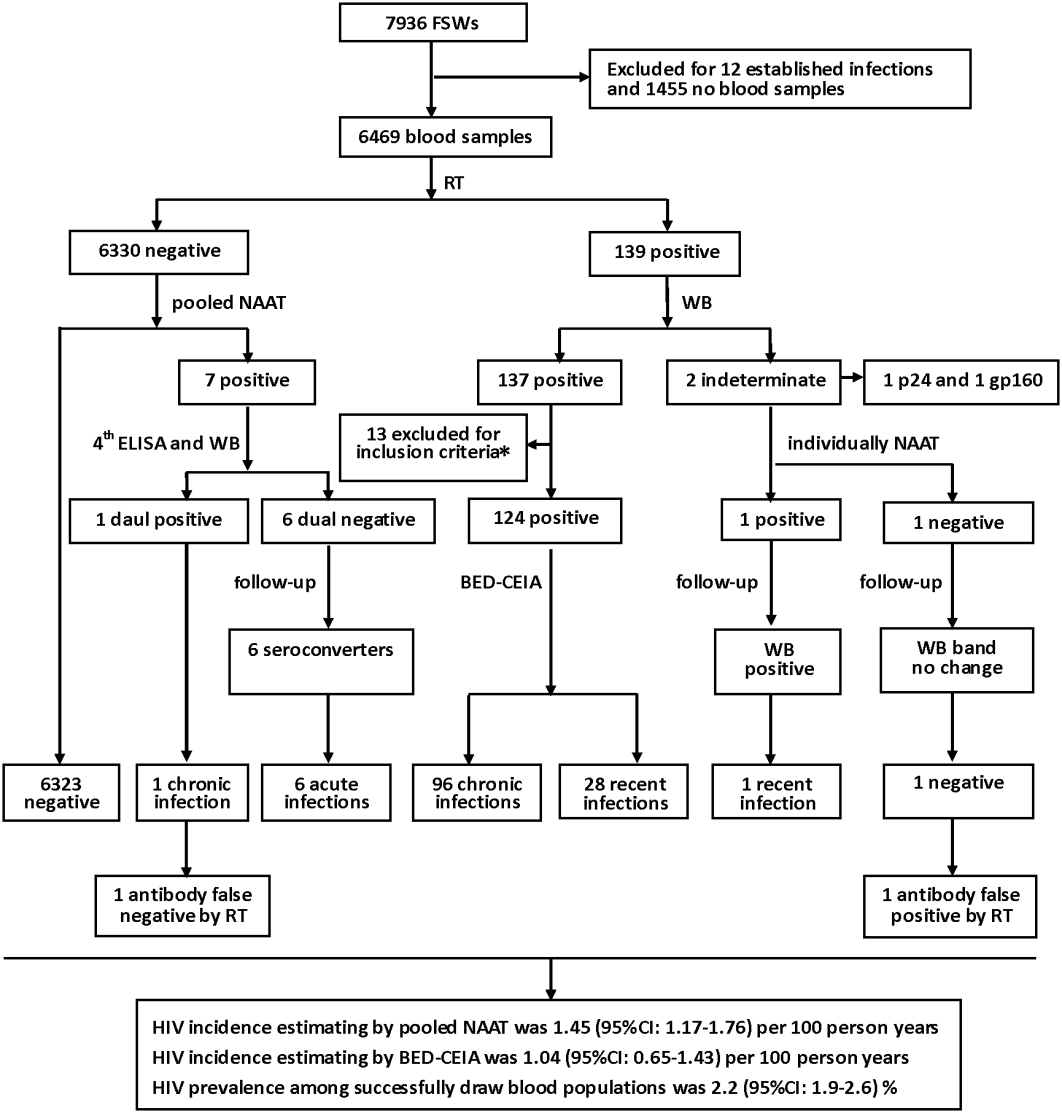
Flow Chart of the Study among FSWs from Low-grade Venues and HIV Infection Detection Process. The numbers of study samples, HIV detection assays and the numbers of different HIV results were shown in the flow chart. HIV incidences were estimated base on the data of pooled NAAT and BED-CEIA respectively. HIV prevalence was calculated based on the numbers of HIV results. Exclusion criteria*: the samples from individuals with a history of antiretroviral treatment (ART) or HIV infections >1 year.

A total of 7936 FSWS were recruited and answered the questionnaires. There were 1455 (18.3%) participants who refused to have blood drawn for HIV infection test, and 12 (0.2%) participants who were already diagnosed of chronic HIV infections. We successfully collected blood samples from 6469 (81.5%) participants, of which 139 (2.1%) were HIV antibody-positive and 6330 (97.9%) were HIV antibody-negative by RT (Figure 2).

All 6330 specimens, which were HIV antibody-negative, were tested by pooled NAAT (Figure 1). These specimens were first divided into 633 intermediate pools and then subdivided into 127 master pools. There were 7 positive master pools and 7 positive intermediate pools detected by pooled NAAT respectively. Of 7 positive intermediate pools, 70 specimens were tested further by NAAT individually for HIV RNA. As a result, seven (0.1%) specimens were found positive for HIV RNA, identifying an additional 5.0% of HIV-infected persons. Therefore, the HIV incidence estimated based on the data of pooled NAAT was 1.45 (95% CI: 1.17–1.76) per 100 person years.

The 7 HIV RNA positive/antibody-negative specimens were tested by the 4^th^ generation ELISA and WB, which identify 1 dual positive specimen and 6 dual negative specimens. The 6 dual negative participants were informed and followed-up to collect blood samples in 3 months. Follow-up analysis by the 4^th^ ELISA and WB showed that all 6 participants had HIV antibody seroconvertion. So, we identified 6 acute HIV infections and 1 chronic HIV infection among 6330 RT negative specimens by applying the pooled NAAT. The laboratory results and follow-up of 7 cases with HIV RNA positive/HIV antibody-negative participants were shown in Table 1.

**Table 1:** The Laboratory Results and Follow-up of 7 Cases with HIV RNA Positive by Pooled NAAT*.

Sample N0.	HIV Ab	HIV Ab/p24 Ag	HIV Ab	Viral load (copies/ml)	HIV Ab (WB, Follow-up)
	RT	4^th^ ELISA	WB		
450126088	Neg	Neg	Neg	16000	Pos
450701015	Neg	Neg	Neg	5900	Pos
450701053	Neg	Neg	Neg	3300000	Pos
450401673	Neg	Neg	Neg	222000	Pos
450924077	Neg	Pos	Pos	2370	no follow-up
450802005	Neg	Neg	Neg	5650	Pos
450802119	Neg	Neg	Neg	6800	Pos

*: Ab: antibody, Ag: antigen, Neg: negative, Pos: positive, Follow-up performed in 3 months.

Among 139 RT positive samples, there were 137 positive and 2 indeterminate by WB. We excluded 13 HIV antibody positive specimens (2 had ART history and 11 diagnosed of HIV infections more than 1 year) from the BED-CEIA testing protocol. The remaining 124 (90.5%) HIV antibody positive specimens tested by BED-CEIA were found to have 96 chronic infections and 28 recent infections. Thus, HIV incidence estimated based on the BED-CEIA data was 1.04 (95% CI: 0.65–1.43) per 100 person years, which was similar to that by the pooled NAAT as just described earlier. Analysis of the 2 WB-indeterminate specimens by NAAT showed that one was positive and the other was negative for HIV RNA. Follow-up confirmatory WB analysis for these 2 participants showed that the one positive for HIV RNA developed a new band of p24 meeting the criteria of WB positive and the other had no HIV-specific bands progressed being classified as WB negative.

Overall, there were 97 chronic infections, 6 acute infections, 29 recent infections and 6324 negative infections among the 6469 participants. The overall prevalence of HIV among FSWs from low-grade venues in Guangxi was 2.2 (95% CI: 1.9–2.6) %.

## Discussion

We have demonstrated that the addition of HIV RNA screening to routine HIV antibody testing among FSWs from low-grade venues could detect acute HIV infections and identified an additional 5% of HIV-infected persons. These findings have important public health implications for the local HIV prevention. HIV infected FSWs were known to greatly increase the spread of HIV-1 infection [27]–[29]. Our improved detection of HIV infection through application of HIV RNA screening could substantially reduce the potential risks for secondary HIV transmission by HIV infected FSWs who are unaware of their HIV infections. A prompt diagnosis of acute HIV infection might prevent the infected FSWs from engaging in high-risk behaviors with uninfected male clients and avoid new HIV infections to occur. But FSWs with acute HIV infection should take counseling regarding risk-reduction strategies such as abstinence and safer sexual behaviors with 100% use of condoms. Studies have also showed that counseling interventions at the time of the receipt of an HIV-positive test result were associated with a significant decrease in risk behavior [27]. We strongly suggest that this strategy should be considered a priority in the prevention program of acute HIV infection. At the same time, tracing source patients and their exposed partners may help characterize the sexual and social networks and facilitate the targeted interventions [30].

Our survey study showed that the overall prevalence of HIV infections among FSWs from low-grade venues in Guangxi was 2.2 (95% CI: 1.9–2.6) %. This HIV prevalence rate was significantly lower than those derived from surveys (8.3–10.3%) performed in neighboring province Yunnan [31], [32], but much higher than the median prevalence (0.6%) among 15 studies in China [5]. Compared with the results of previous surveys among FSWs in Guangxi, the HIV prevalence rate in this study was similar to one in 2005 (2.3%) [7], but higher than the others [8], [9] in 2007 (0.8%) and in 2010 (1.0%).

We also found that the BED-CEIA-derived HIV incidence was 1.04 per 100 person years (95% CI: 0.65–1.43), which was close to the pooled NAAT-derived HIV incidence (1.45 per 100 person years [95% CI: 1.17–1.76]). To our knowledge, this was the first study in China using both BED-CEIA method and pooled NAAT method to test samples from the same subjects and evaluating data from both tests to estimate the HIV incidence among high-risk populations of FSWs. It appeared that the HIV incidence rates in Guangxi and Yunnan were similar. For example, the HIV incidence in Dehong, Yunnan remained relatively stable at 1.3–1.4% between 2004 and 2008 [33]. Similarly, a 1.1% HIV incidence was found in Kaiyuan from studying a longitudinal cohort and a 1.5–1.6% adjusted incidence was obtained from application of BED-CEIA incidence assay [34].

In addition, we found that HIV infections among FSWs from low-grade venues in Guangxi were significantly associated with factors including no fixed living places, drug use, age of over 40 years old, and ethnic of Vietnamese as reported in our previous published article [35]. The associated factors of HIV infections in this study were similar to those in some previous studies in Guangxi [8], [9] and Yunnan [32], [36]. We therefore suggested that future preventions and interventions should be focused on drug-using FSWs and cross-border foreign FSWs.

It is of note that the survey in this study was done in 2011 and thus might not reflect the latest trends of HIV infection in the FSWs. This may likely limit a precise evaluation of the implemented comprehensive measurements in prevention and control of HIV transmission in Guangxi. Technically, the BED-CEIA related parameters, such as the window period (155 days) and the confirmatory ODn value (0.8), may not be the same as those for Chinese HIV subtypes. In the meantime, whether the use of the Chinese HIV subtypes as the positive control in the BED-CEIA assay could lead to misclassification remained to be determined.

One important focus of our present study was identification of the acute HIV infection among the FSWs by using combination of HIV RNA screening with routine HIV antibody tests. Our approach has achieved a higher sensitivity enabling the detection of acute HIV infection, however, at the expense of a bigger volume of each specimen and thus the sacrifice of genotyping the HIV subtype of the newly infected patients. Given that CRF01_AE was reported to be the most prevalent subtype of HIV-1 strain in sexually transmitted infections in Yunnan [10] and has become the dominant subtype of current HIV epidemic in heterosexual transmission populations in Guangxi [11], we predicted that CRF01_AE was very likely the genotype of HIV found in FSWs with acute HIV infections. Therefore, further studies are required to clarify these issues.

In conclusion, we have proved that HIV RNA screening in combination with routine HIV antibody testing significantly improved the detection of HIV infections among FSWs from low-grade venues, giving a more accurate HIV incidence rate in FSWs population in Guangxi. Our study also provided useful baseline data of HIV incidence among this population for targeting local HIV prevention, intervention, monitoring and treatment.
